# Mo-BiVO_4_ Photocatalytically Modified Ceramic Ultrafiltration Membranes for Enhanced Water Treatment Efficiency

**DOI:** 10.3390/membranes14050112

**Published:** 2024-05-14

**Authors:** George V. Theodorakopoulos, Martha Pylarinou, Elias Sakellis, Fotios K. Katsaros, Vlassis Likodimos, George Em. Romanos

**Affiliations:** 1 Institute of Nanoscience and Nanotechnology, National Center of Scientific Research “Demokritos”, 15341 Agia Paraskevi, Greece; e.sakellis@inn.demokritos.gr (E.S.); f.katsaros@inn.demokritos.gr (F.K.K.); 2 Inorganic and Analytical Chemistry Laboratory, School of Chemical Engineering, National Technical University of Athens, Zografou Campus, 9, Iroon Polytechniou Str., 15772 Zografou, Greece; 3 Section of Condensed Matter Physics, Department of Physics, National and Kapodistrian University of Athens, 15784 Athens, Greece; pylarin@phys.uoa.gr (M.P.); vlikodimos@phys.uoa.gr (V.L.)

**Keywords:** Mo-BiVO_4_, ultrafiltration membrane, vis-light, tetracycline, Total Organic Carbon (TOC)

## Abstract

This study highlights the effectiveness of photocatalytically modified ceramic ultrafiltration (UF) membranes in alleviating two major drawbacks of membrane filtration technologies. These are the generation of a highly concentrated retentate effluent as a waste stream and the gradual degradation of the water flux through the membrane due to the accumulation of organic pollutants on its surface. The development of two types of novel tubular membranes, featuring photocatalytic Mo-BiVO_4_ inverse opal coatings, demonstrated a negligible impact on water permeance, ensuring consistent filtration and photocatalytic efficiency and suggesting the potential for maintaining membrane integrity and avoiding the formation of highly concentrated retentate effluents. Morphological analysis revealed well-defined coatings with ordered domains and interconnected macropores, confirming successful synthesis of Mo-BiVO_4_. Raman spectroscopy and optical studies further elucidated the composition and light absorption properties of the coatings, particularly within the visible region, which is vital for photocatalysis driven by vis-light. Evaluation of the tetracycline removal efficiency presented efficient adsorption onto membrane surfaces with enhanced photocatalytic activity observed under both UV and vis-light. Additionally, vis-light irradiation facilitated significant degradation, showcasing the versatility of the membranes. Total Organic Carbon (TOC) analysis corroborated complete solute elimination or photocatalytic degradation without the production of intermediates, highlighting the potential for complete pollutant removal. Overall, these findings emphasize the promising applications of Mo-BiVO_4_ photocatalytic membranes in sustainable water treatment and wastewater remediation processes, laying the groundwork for further optimization and scalability in practical water treatment systems.

## 1. Introduction

Clean water is a critical resource for sustainable development, yet its availability is increasingly challenged by growing demands and the impacts of climate change. Consequently, water reclamation and reuse have been implemented as essential strategies. Nevertheless, the presence of persistent organic pollutants in water poses a significant challenge due to their toxicity and limited biodegradability. In recent years, there has been a growing demand for catalytic oxidation processes in various environmental applications, particularly in the treatment of water and wastewater [1,2,3].

Advanced Oxidation Processes (AOPs) are promising and emerging techniques that have garnered considerable interest from researchers. This is attributed to their high effectiveness in eliminating persistent and resilient pollutants, their non-selective behavior and eco-friendly characteristics, and the fact that they do not produce solid waste. These advancements underscore the substantial progress achieved in catalytic oxidation processes for environmental applications. Amongst a great variety of AOPs, photocatalysis has the capacity to oxidize highly recalcitrant organic compounds, thus constituting an effective solution for wastewater treatment, especially when the target is to recycle industrial water effluents. Titanium dioxide (TiO₂) has emerged as a widely studied material, owing to its exceptional photocatalytic characteristics and low cost. However, due to the inherent limitations of TiO_2_, which are mostly related to its wide band gap and the necessity for UVA radiation, there is the need for new advanced materials that will be effective under solar or visible light radiation.

In this context, bismuth vanadate (BiVO_4_) has attracted significant interest as a promising semiconductor for photocatalytic and photoelectrochemical applications such as the degradation of emerging pollutants and water splitting. This is attributed to its narrow band gap, suitable valence band position, abundance, and cost effectiveness [4]. Nonetheless, its practical activity fails to meet its theoretical photocurrent density under solar light illumination due to rapid charge recombination and slow oxidation kinetics [4,5]. Transition metal ions, such as molybdenum (Mo), have been employed to enhance the performance of BiVO_4_ under solar light [4,5,6]. In our recent study [6], a Mo-BiVO_4_ inverse opal film was deposited onto a fluorine-doped tin oxide (FTO) coated glass substrate. Similar approaches have been reported in literature, where these inverse opal films were applied onto FTO-coated glasses using either dip-coating [7], drop-coating [8], or spin-coating [9] techniques. Additionally, Ta et al. [10] developed a macroporous-structured WO_3_/Mo-doped BiVO_4_ photoanode on titanium microfiber felt.

On the other hand, membrane processes have gained considerable interest in recent years as a proficient approach for water purification and wastewater treatment, owing to their superior efficiency, minimal energy usage, and straightforward operation. Nevertheless, conventional filtration with membranes frequently encounters challenges such as fouling and limited selectivity, which may impair performance over time. To address these issues, researchers have explored diverse methodologies aimed at intensifying membrane processes and enhancing their characteristics. One promising solution resides in the development of photocatalytic membranes, which integrate filtration with advanced oxidation processes. The approach entails the incorporation of photocatalytic materials into membrane structures, leading to the development of photocatalytic modified membranes, which are engineered not only to reject but also to degrade organic contaminants. These innovative membranes represent a cutting-edge technology as they integrate the principles of photocatalysis into membrane structures, providing a multifunctional approach to address issues related to water purification, air treatment, and various environmental remediation applications.

Fundamentally, photocatalytic membranes harness the catalytic power of semiconductor materials immobilized onto or within the membrane matrix to induce photochemical reactions upon exposure to light. Under irradiation with ultraviolet (UV) or visible light, these photocatalysts generate reactive oxygen species (ROS), which can degrade organic pollutants, disinfect microorganisms, and mitigate fouling on the membrane surface. Consequently, the integration of photocatalytic functionality into membrane systems enhances their overall performance by providing an additional layer of active degradation for contaminants. This synergy between membrane filtration and photocatalysis not only improves the removal efficiency of pollutants, but also contributes to the mitigation of fouling issues, prolonging membrane lifespan and reducing maintenance requirements. This makes photocatalytic membranes promising candidates for sustainable and efficient water treatment processes. Several studies have demonstrated the effectiveness of photocatalytic membranes in various water treatment applications [11,12,13,14,15,16]. Moreover, the integration of photocatalytic materials into membrane structures can enhance the selectivity and specificity of membranes for particular contaminants, making them suitable for targeted water treatment applications. In summary, photocatalytic membranes represent a promising approach for sustainable water treatment and wastewater remediation, providing enhanced pollutant removal efficiency, resistance to fouling and improved selectivity. Consequently, these membranes hold great potential for enhancing water treatment efficiency and thus play a crucial role for the development of a more sustainable and robust water supply system.

**Scheme 1:**
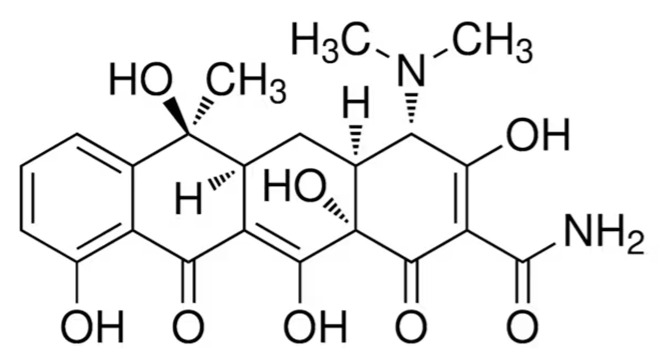
The molecular structure of tetracycline (Formula: C_22_H_24_N_2_O_8_).

Tetracycline (Scheme 1), a broad-spectrum antibiotic with M_w_ = 444.435 g/mol, extensively used in aquaculture, human and veterinary medicine [17,18], is raising concerns due to its persistence in the environment and potential adverse effects on ecosystems [19,20] and human health [21,22]. Daghrir and Drogui [23] reported that following medication, over 70% of tetracycline antibiotics are excreted and released in an active form into the environment through urine and feces from both humans and animals. Furthermore, it has the potential to accumulate within the food chain, leading to toxicity in the microbial community [17], promoting the emergence and spread of antibiotic resistance, posing risks to drinking and irrigation water and disturbing the microbial flora in the human intestine [18]. On the contrary, insufficient wastewater systems are a key factor contributing to the increase in antibiotic concentrations in aquatic sources. Therefore, it can be deduced that its widespread usage, subsequent excretion, and improper disposal methods [18] have led to its detection across various environmental matrices, including surface water, soil, and wastewater treatment facilities. Hence, the imperative development of efficient degradation methods to mitigate its environmental impact cannot be overstated. Photocatalysis has arisen as a promising method for the degradation of tetracycline contaminants in aqueous environments [6,24,25,26,27,28,29]. As aforementioned, this process utilizes photocatalysts to generate reactive oxygen species under irradiation with ultraviolet (UV) or visible light. These ROS, which include hydroxyl radicals (•OH), superoxide radicals (•O_2_^−^), and singlet oxygen (^1^O_2_), are highly reactive and can oxidize organic pollutants into harmless substances.

To the best of our knowledge, this work is the initial approach to applying an Mo-BiVO_4_ coating onto tubular ceramic ultrafiltration (UF) membranes. Specifically, two photocatalytic membranes were developed by a facile method that entailed the deposition of Mo-BiVO_4_ inverse opal coatings on either one or both surfaces of the membranes. This study aimed to investigate the photocatalytic degradation of tetracycline to assess the effectiveness of Mo-BiVO_4_ under UV and visible irradiation for degrading this highly water-soluble antibiotic and preventing its release to the environment without compromising the ultrafiltration characteristics of the membranes.

## 2. Materials and Methods

Two photocatalytically modified membranes were developed by the deposition of photocatalytic Mo-BiVO_4_ inverse opal coatings on the tubular ceramic UF membranes. The first membrane (designated as mem1) was developed by depositing the coating solely on the lumen, while the second one (mem2) featured coatings applied on both the lumen and shell surfaces. This process involved the liquid infiltration of self-assembled polystyrene (PS) opal templates by a complex metal salt precursor.

Monodisperse PS microspheres of 340 nm diameter (Microparticles GmbH, standard deviation SD = 10–11 nm) in the form of 5% solids (*w*/*v*) colloidal dispersions in deionized water were selected as templating spheres, because they provide the optimal light trapping and size-selective performance on photocatalytic organics degradation by BiVO_4_ inverse opal films [6]. Initially, 500 μL of 1.25% diluted aqueous PS sphere suspension was deposited on the cleaned membrane surface, which was kept at 20 °C until the solvent fully evaporated within a few hours, leading to the spontaneous formation of a closely packed opal template. Liquid infiltration was then applied by direct deposition of the amorphous precursor prepared by mixing two solutions, followed by drying for 1 h at 70 °C. The first solution was prepared by mixing 0.485 mmol ammonium vanadate, NH_4_VO_3_ (ACS, 99.0% min, Sigma-Aldrich, St. Louis, MO, USA), 0.015 mmol ammonium molybdate tetrahydrate (NH_4_)_6_Mo_7_O_24_·4H_2_O (BioUltra, ≥99.0%, Sigma-Aldrich, St. Louis, MO, USA), (Mo:V molar ratio 3% [6]), 9 mL of propanol-2 (HPLC, 99.8%) and 0.5 mL of nitric acid (puriss. p.a., 65%) under continuous stirring for 30 min at room temperature, followed by sonication for 5 min and the addition of 0.5 mmol citric acid (anhydrous, ACS, 99.5+%) until dissolution.

The second one was prepared by dissolving 0.5 mmol bismuth(III) nitrate pentahydrate, Bi(NO_3_)_3_·5H_2_O (99.999% trace metals basis) with 0.5 mL acetic acid (glacial, ACS, 99.7+%). The two solutions were mixed and stirred for 30 min. After drying, calcination was performed at 400 °C in order to remove the sacrificial PS matrix and crystallize the Mo-BiVO_4_ inverse opal coating.

Regarding the commercial ceramic UF membranes utilized in this study, they consisted of a macroporous tubular substrate with a pore size of 2 μm, fabricated through the extrusion of α-alumina. This substrate was augmented with two intermediate γ-alumina layers, each with pore sizes of 200 and 70 nm, respectively that were successively formed by slurry coating on the lumen surface of the substrate. Additionally, an ultrafiltration ZrO_2_ layer with a pore size of 3 nm was deposited atop the γ-alumina layer featuring 70 nm pores using sol-gel coating.

The materials’ morphology and structural properties were characterized using a FEI Quanta Inspect scanning electron microscope (SEM) (Eindhoven, The Netherlands) coupled with energy-dispersive X-ray analyzer (EDX) and a FEI Talos F200i scanning transmission electron microscope (TEM) (Eindhoven, The Netherlands) operating at 200 keV, equipped with a windowless energy-dispersive spectroscopy microanalyzer (6T/100 Bruker). The phase composition of the coatings was also investigated by micro-Raman spectroscopy on a LabRAM Soleil Raman microscope (Horiba, Kyoto, Japan) by means of ×5 (ΝA = 0.15) and ×100 (ΝA = 0.9) objectives using a built-in 532 nm laser excitation and rejection filter with a 50 cm^−1^ cut-off on an 1800 lines/mm diffraction grating and a high sensitivity, deep-cooled CCD detector. The optical properties were investigated by diffuse and specular reflectance measurements using a fiber-optic diffuse and a 15° specular reflectance accessory on a Cary 60 UV–vis spectrometer, respectively. A Halon reference and a UV-enhanced Al mirror were used for background determination.

The two types of tubular membranes were developed with the purpose of separately investigating the effect/impact of adsorption and photocatalysis mechanisms on the overall abatement efficiency of the process. The first membrane (mem1) was holding a photocatalytic Mo-BiVO_4_ inverse opal coating that was sprawling on the entire surface of the ZrO_2_ (UF) layer, positioned on the lumen side of the membrane, whereas the second membrane (mem2) was bearing two photocatalytic coatings, one on its shell and the other on its lumen ZrO_2_ surface.

**Scheme 2:**
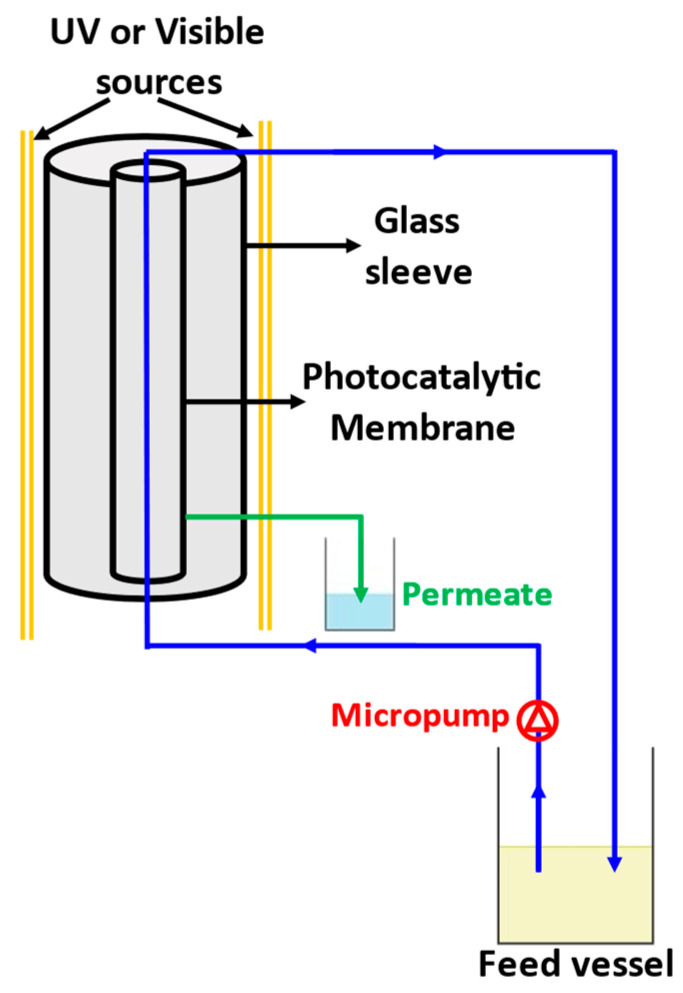
The overall setup for the photocatalytic system employed in the hybrid photocatalytic/ultrafiltration experiments, where the feed inlet, the retentate outlet, and the permeate outlet are distinguished.

To assess the photocatalytic efficiency of the newly developed membranes, tetracycline, a common antibiotic pharmaceutical, was employed as a model pollutant at a concentration of 20 ppm in 500 mL aqueous solutions. In Scheme 2, the overall photocatalytic system employed in the hybrid photocatalytic/ultrafiltration experiments is depicted. The membrane module consisted of a glass tubular shell and stainless-steel top and bottom flanges that held the glass shell and the tubular membrane together and were specially designed to hermetically separate the feed and the permeate side of the membrane. The assembled membrane module was mount vertically at the center of a tubular reflecting enclosure equipped with vis-light and fluorescent UVA sources.

The tetracycline solution underwent continuous recirculation, transferred from the feed vessel to the feed inlet of the membrane module with the aid of a micropump (MG2000F, TCS Micropumps Ltd., Faversham, UK) and returning from the retentate outlet of the module back to the feed vessel. The feed water was conveyed to the lumen side of the tubular membrane, flowing in the direction from the bottom to the top of the module, while the permeate effluent would flow down on the shell surface of the membrane and be collected from the permeate outlet, which was located at the bottom flange of the module in the annular space between the tubular membrane and the glass shell. Hence, the experimental setup depicted in Scheme 1 managed to solely irradiate the shell surface of the tubular membrane during the tangential flow filtration process. This feature allowed us to distinguish between the adsorption and photocatalysis phenomena using the setup for the testing of membranes with and without a photocatalytic Mo-BiVO_4_ inverse opal coating on their shell surface. Throughout all the experiments, samples from both the permeate side and the recirculation vessel were subjected to analysis using a UV–vis spectrophotometer and a TOC analyzer.

In order to ascertain the decomposition rate of the pollutant, samples from the photocatalytic filtration process were analyzed using UV–vis spectroscopy (Cary 100 Conc UV–visible spectrophotometer, Varian Inc., Palo Alto, USA). The photodegradation efficiency (%) was then calculated using the following formula:

(1) $${\Delta C}_{0}\;\left(\%\right)=\left(\frac{C_{0}-\;C}{C_{0}}\right)\times 100\%$$

where C_0_ is the pollutant concentration after adsorption equilibrium (mg/L) and C represents the concentration at any time during the experiment (mg/L).

Furthermore, to clarify the extent of mineralization throughout the photocatalytic filtration campaign, Total Organic Carbon (TOC) measurements were conducted using an online TOC analyzer (BioTector B3500, Hach, Loveland, CO, USA). The carbon removal efficiency (%) was determined using the following equation:

(2) $$\frac{\Delta \left(\mathrm{TOC}\right)}{{\mathrm{TOC}}_{0}}\left(\%\right)=\left(\frac{{\mathrm{TOC}}_{0}-\;\mathrm{TOC}}{{\mathrm{TOC}}_{0}}\right)\times 100\%$$

where TOC_0_ is the initial carbon concentration after adsorption equilibrium (mg/L) and TOC is the final carbon concentration after the photocatalytic run (mg/L).

## 3. Results

### 3.1. Morphological Analysis

**Figure 1:**
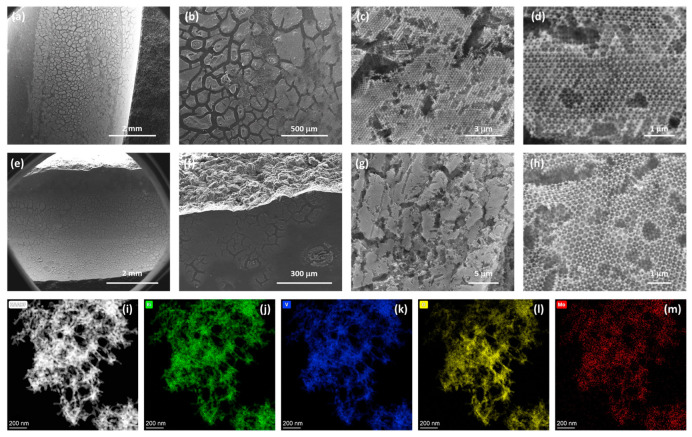
SEM images of Mo-BiVO_4_ coatings on the (**a**–**d**) shell and (**e**–**h**) lumen surfaces of the UF membranes at different magnifications. (**i**) TEM image of the Mo-BiVO_4_ inverse opal and the corresponding (**j**) Bi; (**k**) V; (**l**) O; and (**m**) Mo EDX elemental maps.

Figure 1 shows SEM images on the shell and lumen surfaces of the coated UF membranes at different magnifications. The deposition of inverse opal coatings was clearly observed, corresponding to the (111) planes of an *fcc* lattice of spherical void macropores in the place of the sacrificial PS spheres, surrounded by a solid skeleton that fills the interstitial space. For both membrane surfaces, the coatings consisted of ordered domains extending over approximately 20 μm with a thickness of a few microns separated by cracks, while the average macropore diameter was 210 nm, considerably smaller than the size (340 nm) of the templating PS spheres. These morphological variations resulted from the calcination-induced cracking that persists in crystalline metal oxides via the amorphous-to-crystalline phase transition and the associated volume shrinkage of the inorganic matrix after thermal treatment [30]. Moreover, the primary inverse opal macropores are interconnected through smaller ones (dark circular areas of about 80 nm diameter) creating open windows at the contact points of adjacent spheres after calcination, which are essential for the mass transport of solute species within the pore network. The chemical composition of the Mo-BiVO_4_ coatings is further supported by TEM and EDX analysis, where a homogeneous distribution of Mo species is observed in the corresponding EDX elemental maps along with the Bi, V, and O elements.

### 3.2. Raman Analysis

**Figure 2:**
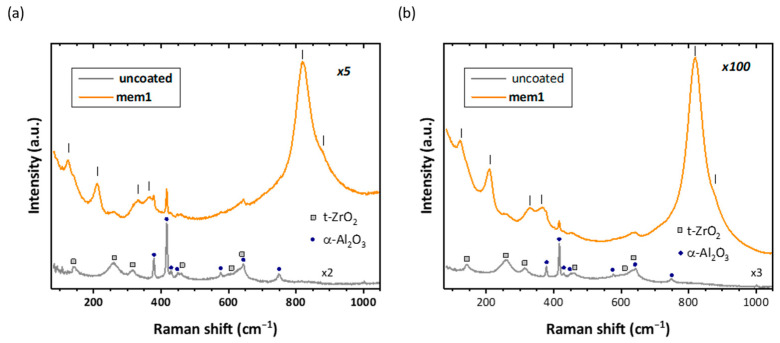
Micro-Raman spectra of the Mo-BiVO_4_ coated membranes in comparison to the bare UF ceramic support using: (**a**) ×5 and (**b**) ×100 microscope objectives at 532 nm. Squares and circles indicate the α-Al_2_O_3_ and t-ZrO_2_ Raman modes, respectively, while vertical lines depict the Mo-BiVO_4_ Raman bands.

The phase composition of the Mo-BiVO_4_ coatings was investigated in comparison to the bare UF membrane support, by micro-Raman spectroscopy at 532 nm. The unmodified ceramic membrane presents two distinct groups of vibrational modes, discriminated by their different spectral widths, as shown in Figure 2a. Specifically, a series of narrow bands are detected at 378, 417, 429, 449, 577, 644, and 750 cm^−1^ that can be identified with the Raman modes of the α-Al_2_O_3_ corundum support [31,32,33] with no contribution of the γ-Al_2_O_3_ intermediate layers that are weak Raman scatterers due to their cubic symmetry [34,35]. In addition, six broad Raman bands are observed at 143, 259, 316, 457, 608 (shoulder), and 640 cm^−1^, which comply favorably with the Raman-active vibrational modes of the tetragonal zirconia phase (t-ZrO_2_) [36,37], arising from the UF ZrO_2_ layer. The intensity of the latter Raman bands appeared to have increased relative to the sharp α-Al_2_O_3_ Raman modes in the corresponding Raman spectra acquired under the ×100 microscope objective (Figure 2b), leading to smaller scattering volume and higher surface sensitivity to the upper UF ZrO_2_ layer compared to the Raman spectra detected by focusing the laser beam on the membrane surface through the ×5 objective.

Micro-Raman spectra of the Mo-BiVO_4_ coated membrane exhibited the characteristic Raman modes of the monoclinic scheelite (*ms*) BiVO_4_ phase with no sign of the tetragonal scheelite or zircon polymorphs [38]. The strong symmetric (ν_s_) V–O stretching mode was observed at 822 cm^−1^ [39], while the monoclinic distortion of the scheelite structure was confirmed by the observation of two distinct Raman bands at 366 and 328 cm^−1^ arising from the symmetric (δ_s_) and antisymmetric (δ_as_) bending modes of the VO_4_ tetrahedra, as well as the two intense external (rotation/translation) lattice modes at 210 and 124 cm^−1^ [40]. The introduction of Mo^6+^ dopants substituting for V^5+^ cations in BiVO_4_ was traced by the observation of an additional weak shoulder at about 880 cm^−1^ [5] related to the corresponding MoO_4_ stretching vibration [6,41]. In addition, the ν_s_ band shifted to lower frequencies compared to unmodified BiVO_4_ inverse opal films (829 cm^−1^), while the δ_s_ and δ_as_ VO_4_ bending modes approached each other for the Mo-BiVO_4_, indicating the averaging of the VO_4_ tetrahedral deformation and the reduction of the monoclinic lattice distortion towards the tetragonal phase [6]. Micro-Raman spectra acquired by focusing the laser beam through the ×100 objective (Figure 2b), resulted once more in significantly enhanced Raman modes for the Mo-BiVO_4_ coating.

### 3.3. Optical Properties

**Figure 3:**
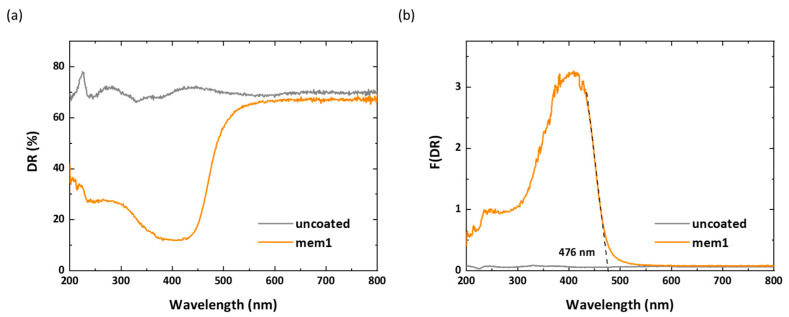
(**a**) Diffuse reflectance (DR%) spectra and the (**b**) corresponding Kubelka–Munk transforms F(DR) for the Mo-BiVO_4_ coated membranes in comparison to the bare UF ceramic support.

The optical properties of the Mo-BiVO_4_ coated membranes were investigated by diffuse reflectance (DR%) measurements in comparison to the bare UF ceramic support (Figure 3a). A marked decrease in DR% is observed below 500 nm for the coated membranes, arising from the electronic band gap absorption of the Mo-BiVO_4_ layer, in contrast to the high reflectance DR% of the wide band gap t-ZrO_2_/Al_2_O_3_ ceramic support [41,42]. An absorption edge of about 476 nm is derived from the Kubelka–Munk transform F(DR) of the DR% spectra for the Mo-BiVO_4_ coatings (Figure 3b), in agreement with previous works on BiVO_4_ inverse opals [6,43], while weak absorption bands are observed at about 250 nm, most probably arising from defect states in the t-ZrO_2_ [44] or Al_2_O_3_ [45] membrane layers.

In our previous study [6], the obtained E_g_ value (2.56 eV) for the Mo-BiVO_4_ metal oxide confirmed the existence of shallow defect states, as evidenced by a Fermi level shift toward the conduction band minimum E_CBM_. This observation aligns with the formation of donor states.

### 3.4. Study of Water Permeance of Photocatalytic Membranes

To enable a UF membrane with a 3 nm pore size (1000 Da M_W_ cut off) to effectively abate the presence of small solutes like tetracycline (M_W_ of 400 Da), alternative mechanisms beyond mere size exclusion are essential. These mechanisms include Donnan exclusion [46,47], where charged molecules are repelled due to the membrane surface and solute carrying similar charges, as well as the strong adsorption of polar molecules onto the membrane’s surface. In our specific case, the presence of a photocatalytic coating (Mo-BiVO_4_) that can be activated through irradiation during the ultrafiltration process, introduces a third potential mechanism, that of photocatalysis.

The initial investigation focused on assessing the impact of the Mo-BiVO_4_ coating on the water permeance of the membranes. The water permeance Pe (L/m^2^·h·bar) is a membrane property calculated from the following equation [48]:

(3) $$\mathrm{Pe}\;=\frac{F}{P\;\times \;S}$$

where F (mL/min) is the water flux through the membrane, P (bar) is the transmembrane pressure (TMP), and S (m^2^) is the surface of the membrane, which, in our case, coincides with the photocatalytic lumen surface of it.

**Figure 4:**
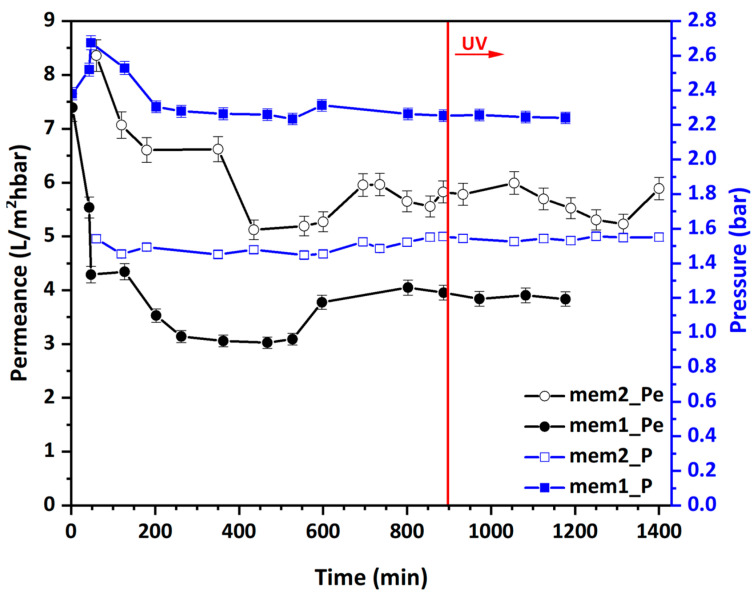
Water permeance of the two Mo-BiVO_4_ photocatalytic membranes. Mem1 is the tubular membrane with one Mo-BiVO_4_ coating on the lumen side and mem2 is the membrane with two Mo-BiVO_4_ coatings on the shell and lumen sides. All experiments were performed with ultrapure water (Milli-Q, 18 MΩ·cm). The red vertical line indicates the time when the UV lamps were activated.

This comparison involved evaluating the performance of membranes with a single Mo-BiVO_4_ coating (mem1) against those with two Mo-BiVO_4_ coatings (mem2). The results of this comparison are depicted in Figure 4.

The almost identical permeance values unveil that the Mo-BiVO_4_ coating predominantly covered the external surface of the membrane without affecting the pores of the UF layer located beneath. Additionally, the steady state water permeance values, ranging from 3 to 6 Lm^2^h/bar, are typical for UF membranes with 3 nm pores, indicating that the pores of the ZrO_2_ UF layer were not plugged by the deposited Mo-BiVO_4_ photocatalyst.

It is also interesting that mem2, despite having two photocatalytic coatings instead of one, exhibits a slightly higher steady state permeance compared to mem1. Furthermore, the additional coating of mem2 is deposited on the shell surface exposed by the macroporous α-Al_2_O_3_ substrate of 2 μm pore size [49]. Due to the much larger pore size of the α-Al_2_O_3_ substrate as compared to the PS spheres (340 nm), the latter can be inserted into the macropore cavities. Consequently, the Mo-BiVO_4_ amorphous phase is also formed inside the macroporous cavities, enveloping the encapsulated PS spheres. The fact that mem2 preserves a high permeance value shows that during calcination, a highly porous inverse opal Mo-BiVO_4_ structure is formed inside the cavities of the substrate, characterized by highly interconnected macropores. Permeance measurements were also conducted during UV irradiation of the shell surface and demonstrated no notable water flux differences compared to those obtained in dark conditions.

**Figure 5:**
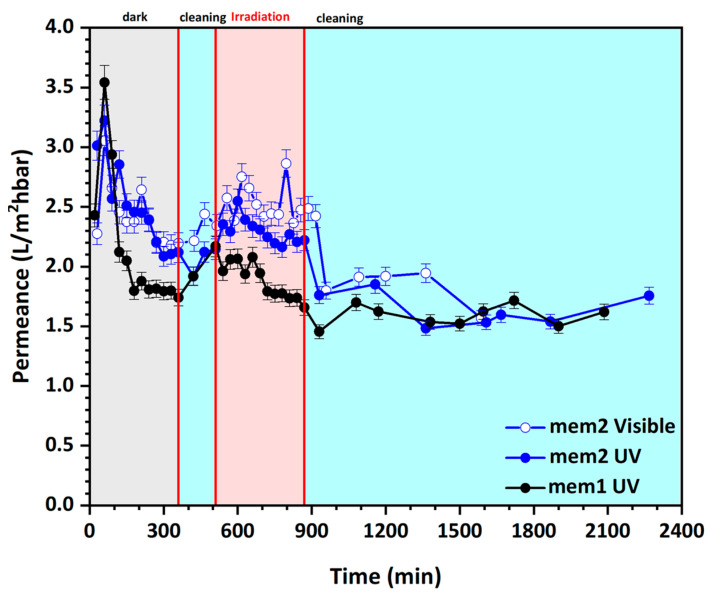
Permeance evolution over time for the two Mo-BiVO_4_ photocatalytic membranes during photocatalytic experiments involving an aqueous solution of tetracycline, including ultrapure water cleaning stages. Dark conditions, ultrapure water cleaning stages, and irradiation conditions (UV or vis-light) are denoted by shaded grey, light cyan, and light red areas, respectively.

The plots in Figure 5 compare the permeance evolution of the two membranes during the experiments with the tetracycline solution. Notably, both membranes exhibit significantly reduced permeance compared to the experiment with pure water (Figure 4), indicating substantial adsorption of tetracycline onto the membrane surface. This is further supported by recent literature indicating that tetracycline can be adsorbed onto bismuth vanadate [6,50], zirconia [51], and/or alumina [52,53]. It is worth highlighting that UV irradiation did not have a profound impact on water permeance and the same applies to the intermediate cleaning stages employing ultrapure water (Milli-Q, 18 MΩ·cm), as depicted by the shaded light cyan areas of Figure 5.

### 3.5. Photocatalytic Efficiency

**Figure 6:**
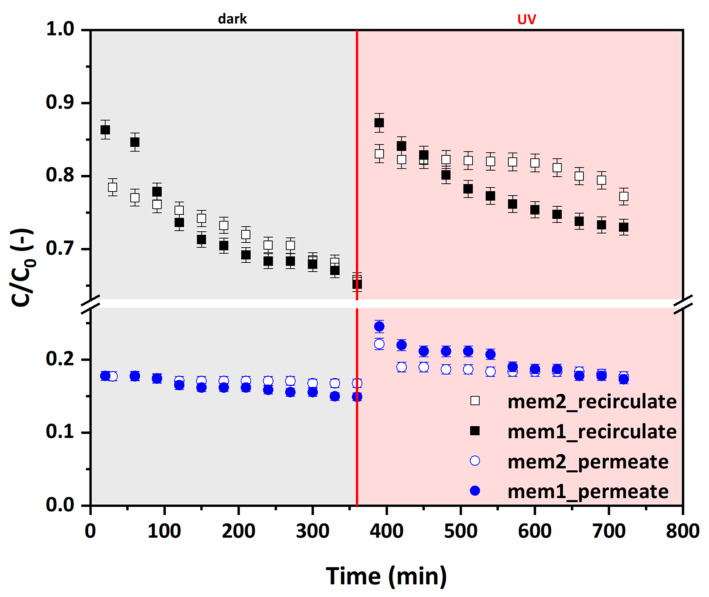
Normalized tetracycline concentration over time for the two Mo-BiVO_4_ photocatalytic membranes (mem1 and mem2) in the permeate effluent and in the recirculation vessel during the experiments under dark and UV conditions. Dark conditions and UV irradiation periods are indicated by shaded grey and light red areas, respectively.

Figure 6 depicts the tetracycline concentration observed in samples collected from both the permeate effluent and the recirculation vessel throughout the experiments. These concentrations are normalized with those of the feed solution. The results of the experiments in dark conditions indicate the great efficiency of both membranes to remove tetracycline from water, which is, however, attributed to the strong adsorption capacity of Mo-BiVO_4_. Notably, there is no evidence of tetracycline rejection, as demonstrated by the absence of higher solute concentrations in the recirculation vessel compared to the feed solution, which would gradually increase over the experiment’s duration. As such, the sudden attenuation of the concentration upon the switching on of the UV lamps is attributed to increased surface temperature on the membrane, leading to sudden and rapid desorption of the tetracycline molecules. Furthermore, the lower tetracycline concentration observed at the permeate side of mem2 during UV irradiation compared to mem1 suggests the occurrence of photocatalysis. This is because mem2 features an additional photocatalytic layer on its shell surface, which is irradiated during the filtration experiment. It could be claimed that the better performance of mem2 is due to enhanced adsorption caused by the higher Mo-BiVO_4_ content (i.e., more Mo-BiVO_4_ leads to higher adsorbance). However, if adsorption was solely responsible for the improved performance, mem2 should also exhibit lower tetracycline concentrations at the permeate effluent even in dark conditions, which is not evident in the obtained results presented in Figure 6.

**Figure 7:**
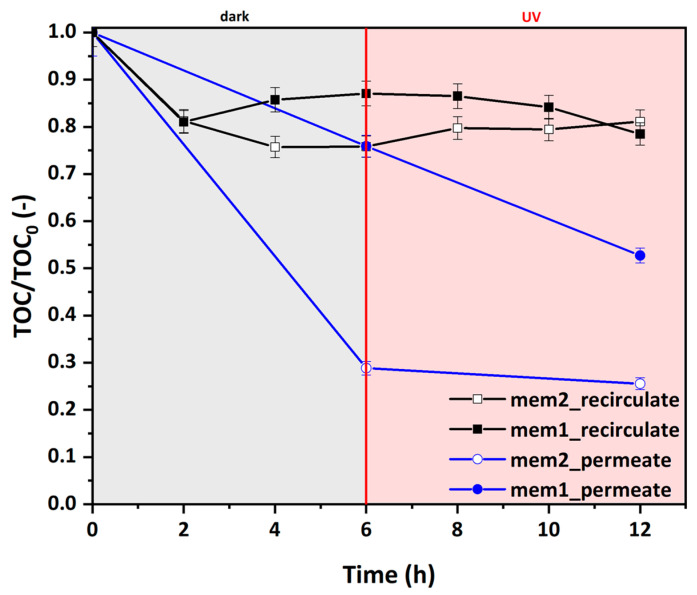
Evolution of the total carbon (TOC) content normalized to the initial TOC_0_ for the two Mo-BiVO_4_ photocatalytic membranes in the permeate effluent and in the recirculation vessel during the experiments under dark and UV conditions. Dark conditions and UV irradiation periods are indicated by shaded grey and light red areas, respectively.

The results from the TOC analysis conducted on selected samples (depicted in Figure 7), when compared with the respective spectroscopic analysis results, indicate either the complete elimination of the solute from the solution due to adsorption or its complete photocatalytic degradation, with no discernible production of any intermediates.

**Figure 8:**
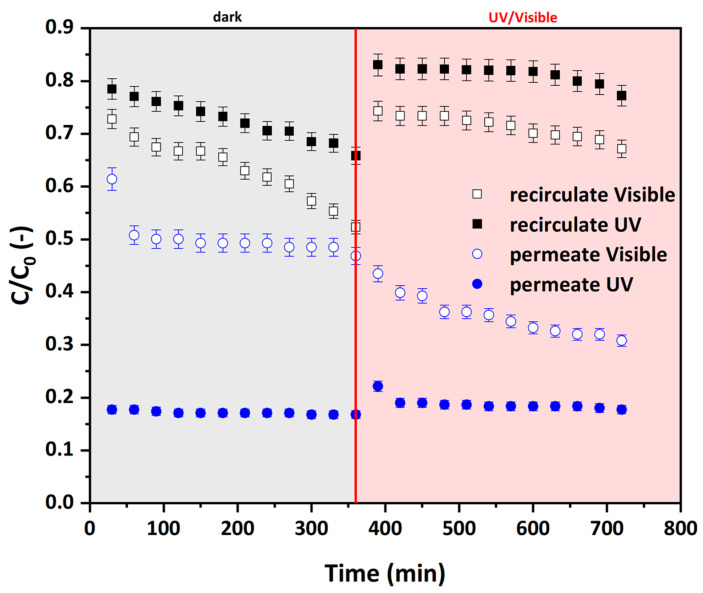
Comparison of the normalized tetracycline concentration over time between the permeate effluent and in the recirculation vessel during experiments conducted in both dark conditions and under irradiation (UV and vis-light) for mem2. Dark conditions and UV/visible irradiation periods are indicated by shaded grey and light red areas, respectively.

Elaboration of the capacity of the externally deposited Mo-BiVO_4_ coating (mem2) to photocatalytically degrade tetracycline under visible light was achieved through a second cycle of experiments. The cycle initially involved the cleaning of mem2 with ultrapure water, followed by recirculation of the tetracycline solution in dark conditions and subsequently under vis-light radiation. The results are depicted in Figure 8, in comparison with the results obtained in the first experimental cycle (dark–UV). Firstly, regarding the experiment under dark conditions compared to that of the first cycle, it was noted that in this second cycle, mem2 was much more efficient in removing the pharmaceutical from the recirculate and less efficient in removing it from the permeate. This behavior is attributed to both the lower concentration of tetracycline in the feed solution used in this second cycle (10 ppm compared to 20 ppm) and the insufficient cleaning of the shell surface. Given that adsorption is the prevailing tetracycline-abatement mechanism in dark conditions and that the lumen side of the membrane is effectively cleaned due to the high velocity of the flowing water stream, it can be stated that the higher efficiency is due to the lower concentration of tetracycline. On the other hand, the cleaning of the shell surface is achieved through the formation of a permeating water layer that slips down the surface at low velocity. Hence, when cleaning is not effective, this results in surface adsorption sites that remain saturated and thus do not contribute to the removal of tetracycline. Notably, the Mo-BiVO_4_ coating deposited on the shell surface of mem2 also had the capacity to photocatalytically degrade tetracycline under vis-light radiation. Despite that, a steady state was not established during the six hours on stream, and importantly, the concentration of tetracycline in the permeate effluent had already dropped by 35% from the moment that the vis-light sources were switched on.

Moreover, concerning the permeance of mem2 (Figure 5) under vis-light exposure, it demonstrates a similar or slightly increased permeance compared to its UV condition. It is worth noting that despite fluctuations, both membranes exhibit consistently stable permeance levels throughout the observed period, underscoring the robustness of the photocatalytic coatings.

**Figure 9:**
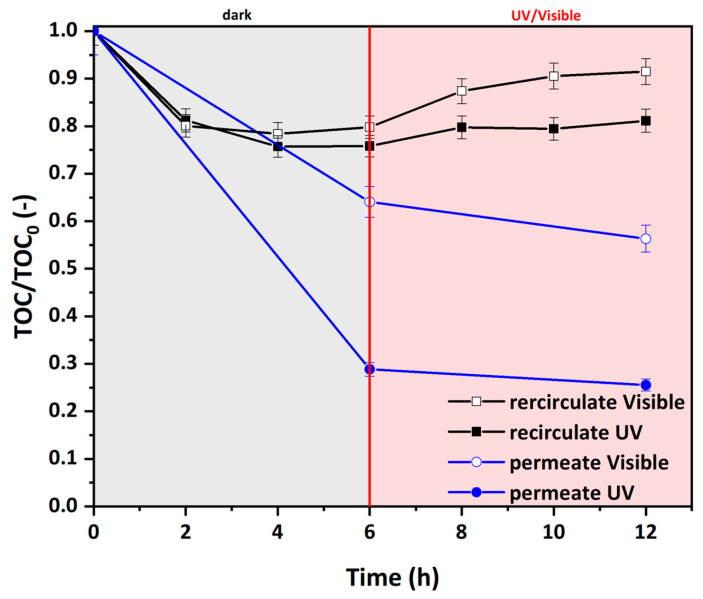
Removal of total organic carbon (TOC) content over time normalized to the initial TOC_0_ for mem2 under dark and UV/visible conditions. Dark conditions and UV/visible irradiation periods are presented by shaded grey and light red areas, respectively.

In addition, the TOC analysis conducted on selected samples (Figure 9) revealed that even in the case of visible light radiation, a significant fraction of the photocatalytically degraded tetracycline molecules is completely mineralized, reaching levels of up to 44%.

## 4. Conclusions

In this study, the development of photocatalytic membranes by depositing Mo-BiVO_4_ inverse opal coatings onto tubular ceramic UF membranes was successfully achieved. Morphological analysis through SEM and Raman spectroscopy confirmed the successful synthesis of Mo-BiVO_4_ coatings. Water permeance evaluation showed the negligible impact of Mo-BiVO_4_ coatings on membrane permeability, with both membranes exhibiting comparable permeance values. This confirms that the coatings predominantly cover the external surface of the membrane without affecting pore structures, preserving steady state water permeance values typical of UF membranes. Adsorption studies indicated a significant reduction in permeance during experiments with tetracycline solution, with mem2 demonstrating an enhanced adsorption compared to that of mem1.

Efficient tetracycline removal (up to 83% in permeate stream) was observed in both membranes, which was attributed to the strong adsorption capacity of Mo-BiVO_4_. UV irradiation induced the rapid desorption of tetracycline molecules due to increased surface temperature. Mem2 exhibited lower tetracycline concentrations during UV irradiation compared to mem1, suggesting enhanced photocatalytic activity. In addition, the photocatalytic degradation of tetracycline under vis-light radiation demonstrated the potential of the developed photocatalytic membranes to effectively degrade contaminants even under different light conditions. TOC analysis showed either complete removal or complete photocatalytic degradation of the solute without the production of any intermediates. Even with vis-light exposure, a significant fraction of tetracycline is fully mineralized, reaching levels as high as 44%. In summary, this study underscores the potential of Mo-BiVO_4_ photocatalytic membranes for the efficient removal of organic contaminants from water, suggesting promising applications in sustainable water treatment and wastewater remediation processes.

## Data Availability

The raw data supporting the conclusions of this article will be made available by the authors on request.
